# Apoptosis Induction, a Sharp Edge of Berberine to Exert Anti-Cancer Effects, Focus on Breast, Lung, and Liver Cancer

**DOI:** 10.3389/fphar.2022.803717

**Published:** 2022-01-27

**Authors:** Yi Zhu, Na Xie, Yilu Chai, Yisen Nie, Ke Liu, Yufei Liu, Yang Yang, Jinsong Su, Chuantao Zhang

**Affiliations:** ^1^ Department of Respiratory Medicine, Hospital of Chengdu University of Traditional Chinese Medicine, Chengdu, China; ^2^ College of Pharmacy, Chengdu University of Traditional Chinese Medicine, Chengdu, China; ^3^ State Key Laboratory of Southwestern Chinese Medicine Resources, Innovative Institute of Chinese Medicine and Pharmacy, Chengdu University of Traditional Chinese Medicine, Chengdu, China

**Keywords:** berberine, bioavailability, ADMET, anticancer effects, apoptosis induction

## Abstract

Cancer is the leading cause of death and one of the greatest barriers to increased life expectancy worldwide. Currently, chemotherapy with synthetic drugs remains one of the predominant ways for cancer treatment, which may lead to drug resistance and normal organ damage. Increasing researches have suggested that apoptosis, a type of programmed cell death, is a promising way for cancer therapy. Furthermore, natural products are important sources for finding new drugs with high availability, low cost and low toxicity. As a well-known isoquinoline alkaloid, accumulating evidence has revealed that berberine (BBR) exerts potential pro-apoptotic effects on multiple cancers, including breast, lung, liver, gastric, colorectal, pancreatic, and ovarian cancers. The related potential signal pathways are AMP-activated protein kinase, mitogen-activated protein kinase, and protein kinase B pathways. In this review, we provide a timely and comprehensive summary of the detailed molecular mechanisms of BBR in treating three types of cancer (breast, lung and liver cancer) by inducing apoptosis. Furthermore, we also discuss the existing challenges and strategies to improve BBR’s bioavailability. Hopefully, this review provides valuable information for the comprehension of BBR in treating three types of cancer and highlight the pro-apoptotic effects of BBR, which would be beneficial for the further development of this natural compound as an effective clinical drug for treating cancers.

## Introduction

Cancer is one of the leading causes of death worldwide with estimated 19.3 million new cases and approximately 10 million deaths in 2020 (gco.iarc.fr; Sung et al., 2021). According to the Global Cancer Observatory, lung cancer is the second most diagnosed cancer and the leading cause of cancer mortality, and it is also the leading cause of death in men. In 2020, 2.2 million new lung cancer cases and 1.8 million deaths were predicted, accounting for 18% of all cancer-related deaths. Among women, lung cancer is the principal cause of cancer-related death in many countries, such as most cities in Eastern Europe, West Asia, East Asia (China), and Southeast Asia (gco.iarc.fr). The incidence rate is generally higher in young women than in young men, which cannot be explained by smoking (Fidler-Benaoudia et al., 2020). Breast cancer is the most commonly diagnosed cancer and the most common cause of death in women. In 2020, approximately 2.3 million women were newly diagnosed with breast cancer and 684,996 women with breast cancer died (gco.iarc.fr). Although the incidence and mortality of breast cancer in high-income countries have been declining, those in low- and middle-income countries have been increasing (Lortet-Tieulent et al., 2020). Globally, the incidence of breast cancer is still on the rise, and there has been a 3.1% annual rate of increase in global breast cancer incidences beginning with 641,000 cases in 1980 to over 1.6 million in 2010 (Bray et al., 2015). Liver cancer was the sixth most commonly diagnosed cancer in the world in 2020 and the third leading cause of cancer-related deaths, with approximately 906,000 new cases and 830,000 deaths. The morbidity and mortality rate of liver cancer in men is two to three times that of women worldwide (gco.iarc.fr). Liver cancer progresses rapidly unless diagnosed early, and the patient’s odds are very low (Gravitz, 2014). Currently, the common treatment methods for cancer include radiotherapy, chemotherapy, and surgery; as research progresses, targeted therapy (Mollaei et al., 2019; Thakkar et al., 2020; Song et al., 2021), immunotherapy (Anderson et al., 2021; Myers and Miller, 2021) and metabolic therapy (Park et al., 2020) have also been used to treat various types of cancer. However, despite the advances that have been made in therapeutic approaches, these treatments may lead to chemotherapy resistance (Zhang et al., 2019d), cancer development (Zhao et al., 2020) and normal organ damage (Zhang et al., 2020b). Owing to socio-economic development, population aging, and lifestyle changes, the incidence of cancer continues to rise worldwide, necessitating the search for safe and effective therapeutic drugs (Sung et al., 2021).

Various natural products have been proven to have anticancer effects and the potential to be effective in cancer treatment (Peng et al., 2015; Wu et al., 2015; Luo et al., 2019a). For example, curcumin can inhibit cancer development and proliferation (Giordano and Tommonaro, 2019), and baicalin promotes apoptosis in pancreatic cancer (Huang et al., 2019b). Natural products are believed to be safer than chemical products (Hou et al., 2021; Xu et al., 2021), as they kill cancer cells without affecting normal cells (El Khalki et al., 2020). Owing to their high availability, low cost, and low toxicity, the application of natural products in cancer therapy has been recommended (Anwanwan et al., 2020). Berberine (BBR) is a well-known isoquinoline alkaloid with multiple biological effects that has been used in medicine for many years (Habtemariam, 2020). It can be easily obtained from medicinal plants and can be synthesized (Kong et al., 2004; Huang et al., 2011). BBR has attracted attention because of its low cytotoxicity and wide variety of pharmacological effects, including antiviral, hypoglycemic, anti-inflammatory, hypotensive, hypolipidemic, and anticancer activities (Feng et al., 2019; Wang et al., 2019; Samadi et al., 2020; Song et al., 2020; Warowicka et al., 2020).

**FIGURE 1:**
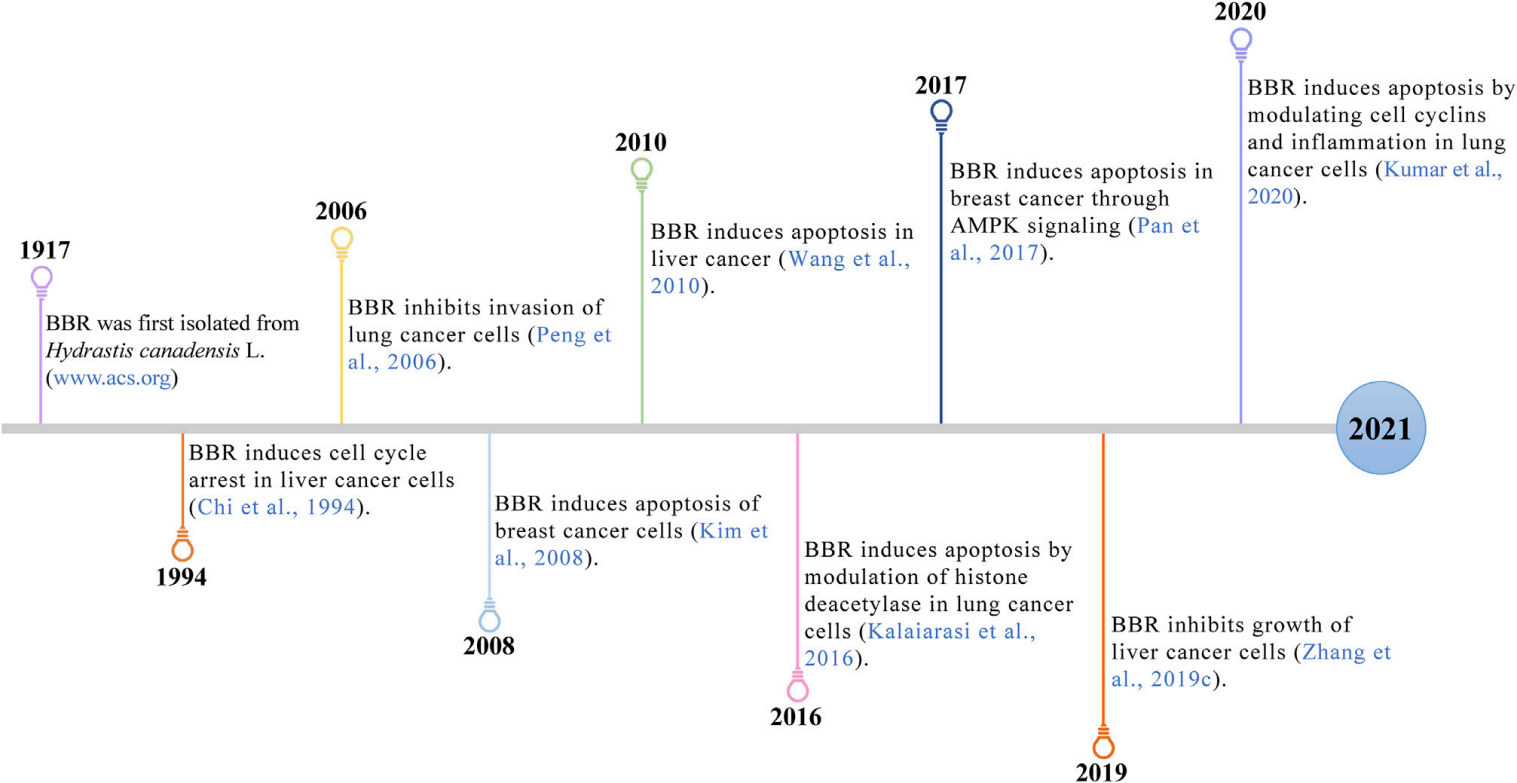
Timeline: Continuous evidences of BBR anticancer effects on three cancers.

Recently, BBR, a well-known natural alkaloid from the *Coptis chinensis* Franch. has shown good potential anticancer effects via the inhibition of cell proliferation, metastasis and invasiveness through apoptosis. The involved mechanisms were related to the regulation of the AMP-activated protein kinase (AMPK), mitogen-activated protein kinase (MAPK), and protein kinase B (AKT) pathways (Figure 1). In this review, we offer a comprehensive summary of the mechanisms of BBR in treating breast, lung and liver cancer, providing a valuable reference for future research.

## Physicochemical Properties of BBR

**FIGURE 2:**
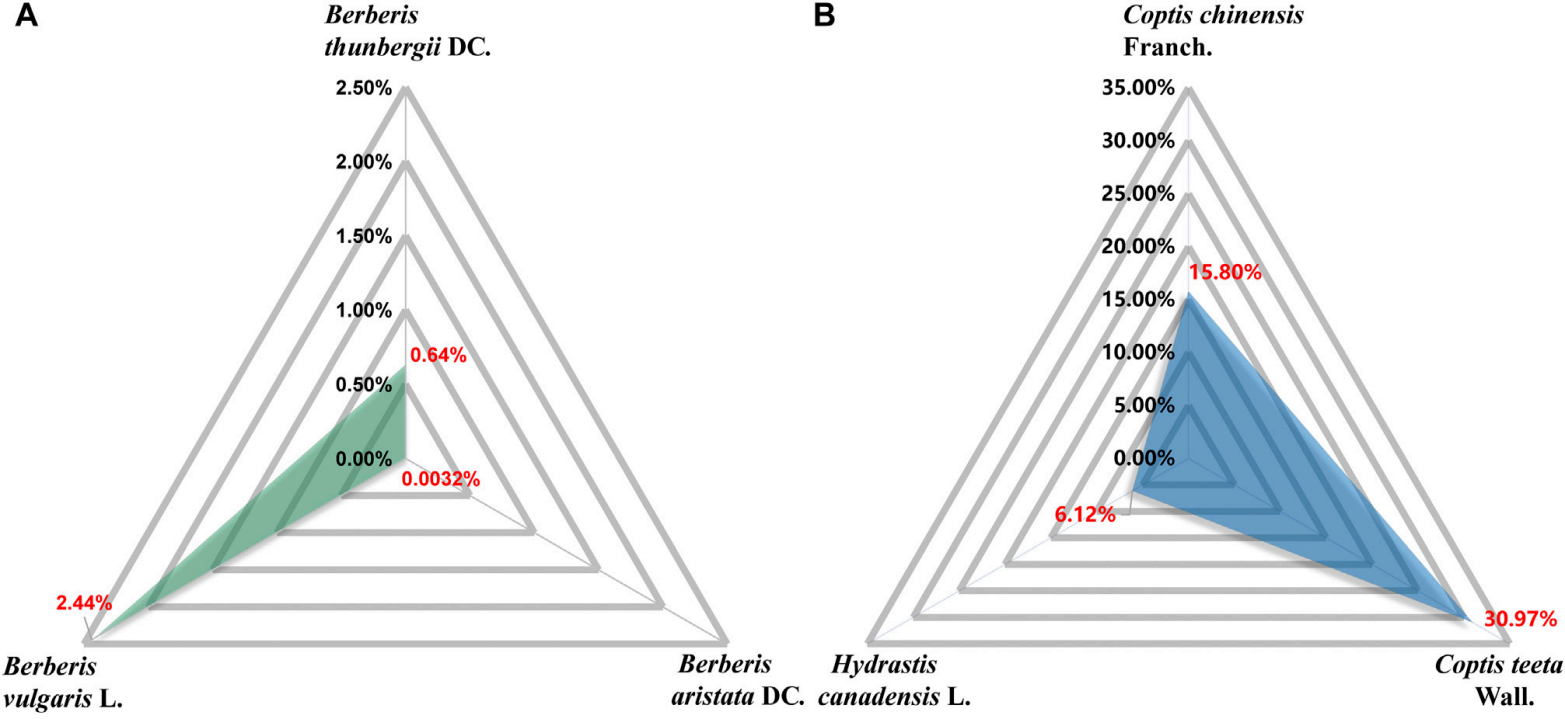
Content of BBR in corresponding plant parts. *Berberis vulgaris* L. dry root: 2.44% (Ahmad et al., 2019). *Berberis thunbergii* DC.: 0.64% (Och et al., 2017). *Berberis aristata* DC. stem bark: 0.0032% (Khan et al., 2020). *Hydrastis canadensis* L. root: 6.12% (Ettefagh et al., 2011). *Coptis chinensis* Franch. water extracts: 15.80% (Roh et al., 2020). *Coptis teeta* Wall. rhizome: 30.97% (Goswami et al., 2019).

BBR (C_20_H_18_NO_4_), with a molecular weight of 336.36 g/mol, is a quaternary amine isoquinoline alkaloid. It is yellow, odorless, and has a very bitter taste. BBR has a wide range of sources, such as *Coptis chinensis* Franch., *Phellodendron chinense* C. K. Schneid., *Berberis vulgaris* L., *Hydrastis canadensis* L., *Arcangelisia flava* (L.) Merr., *Berberis aquifolium* Pursh, and *Berberis aristata* DC. (Peng et al., 2004; Wang et al., 2004) (Table 1). *Berberis vulgaris* L. is the most widely distributed natural source of BBR, containing approximately 2.44% BBR (Ahmad et al., 2019) (Figure 2). BBR is slightly soluble in water, ethanol, or methanol, readily soluble in hot water and hot ethanol, and hardly soluble in organic solvents such as benzene, chloroform and acetone. The salt form is relatively soluble, especially in the sulfate and phosphate forms (Battu et al., 2010; Wang et al., 2017c; Guo and Kang, 2018). Methanol, ethanol, and aqueous or acidified methanol and ethanol are the most commonly used extraction solvents (Teng and Choi, 2014). BBR is sensitive to decomposition under high-temperature and light conditions, and it should be kept at a proper temperature and away from light (Neag et al., 2018).

**TABLE 1:** The plant sources of BBR.

Family	Plant source	Used part	Detection method	Ref.
Ranunculaceae	*Coptis chinensis* Franch.	Rhizome	UHPLC-ESI-MS/MS	Liu et al. (2016)
		Root	HPLC	Roh et al. (2020)
	*Coptis deltoidea* C. Y. Cheng and P. K. Hsiao	Rhizome	UHPLC-ESI-MS/MS	Liu et al. (2016)
	*Coptis teeta* Wall.	Root	HPLC	Liu et al. (2020a)
		Rhizome	HPTLC	Goswami et al. (2019)
	*Hydrastis canadensis* L.	Rhizome, root	LC-MS, HPLC	Ettefagh et al. (2011); Mandal et al. (2020)
	*Coptis japonica* (Thunb.) Makino	Rhizome	HPLC	Min et al. (2006)
Berberidaceae	*Berberis vulgaris* L.	Root	HPLC	Ahmad et al. (2019)
	*Berberis koreana* Palib.	Stem	HPLC	Lee et al. (2010)
	*Berberis thunbergii* DC.	Leaf	HPLC-MS	Fernández-Poyatos et al. (2019)
		—	UPLC-MS/MS	Och et al. (2017)
	*Berberis aristata* DC.	Stem bark	HPTLC	Khan et al. (2020)
	*Berberis asiatica* Roxb. ex DC.	Root, stem bark	HPLC	Andola et al. (2010)
	*Berberis aquifolium* Pursh	Stem bark	Spectrophotometry	Slobodníková et al. (2004)
	*Mahonia bealei* (Fort.) Carr.	Stem	HPLC, qNMR	Wang et al. (2015b)
		—	SFC	Huang et al. (2019c)
	*Berberis aetnensis* C. Presl	Root	HPLC	Campisi et al. (2014)
Rutaceae	*Phellodendron chinense* C. K. Schneid.	Bark	TLC	Zhou and Gu. (1995)
	*Phellodendron amurense* Rupr.	—	HPLC	Wang et al. (2015a)
Papaveraceae	*Chelidonium majus* L.	—	UPLC	Gu et al. (2010)

Note: UHPLC-ESI-MS/MS: ultra-high performance liquid chromatography-electrospray ionization tandem mass spectrometry. qNMR: quantitative nuclear magnetic resonance spectroscopy. HPLC: high-performance liquid chromatography. SFC: supercritical fluid chromatography. HPTLC: high-performance thin-layer chromatography. TLC: thin layer chromatography. UPLC: ultra-performance liquid chromatography. MS: tandem mass spectrometry. -: no relevant data.

## The ADMET Properties of BBR

**FIGURE 3:**
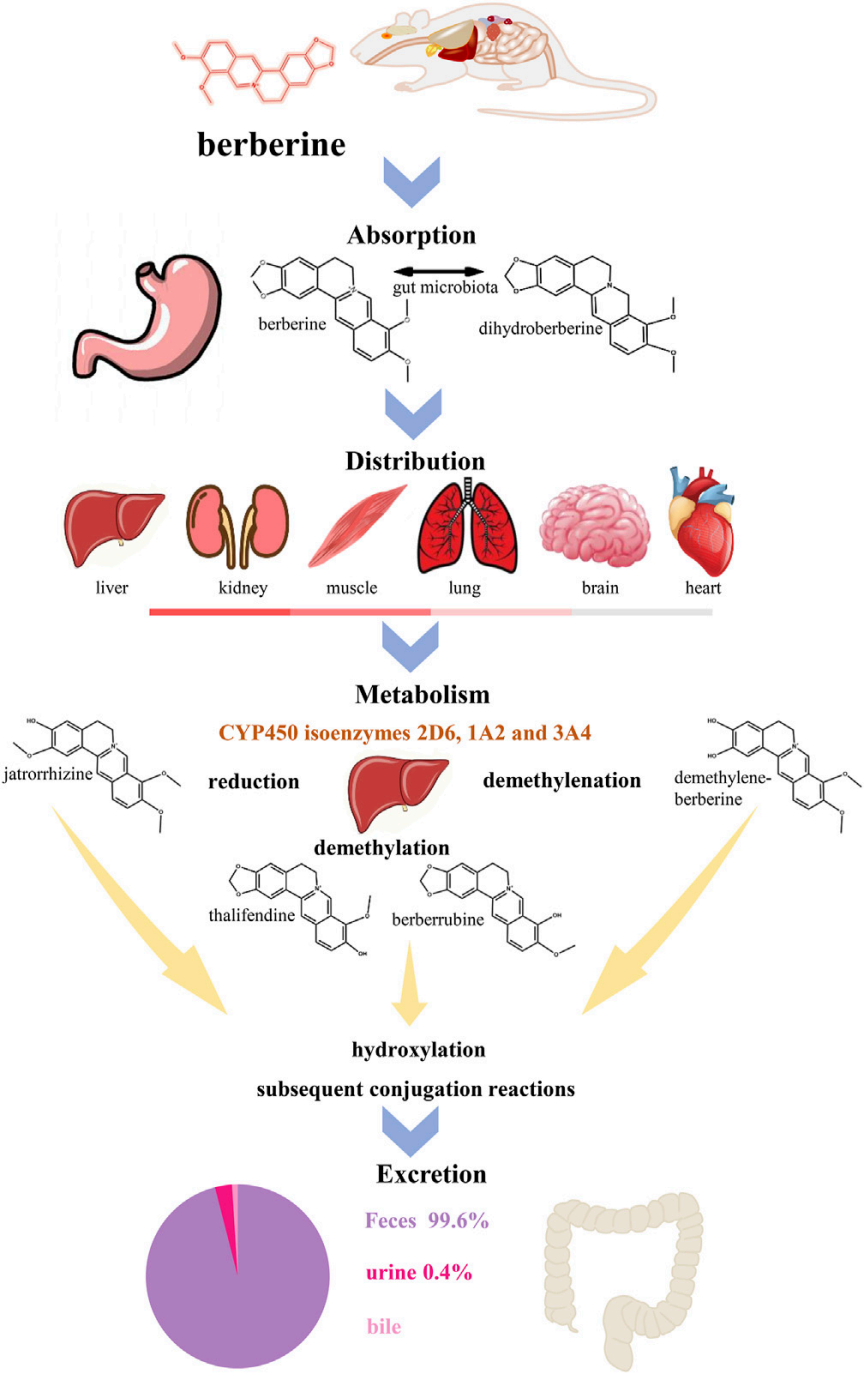
The ADME process of BBR in rats. BBR has a poor oral bioavailability which is below 1% (Habtemariam, 2020). After oral administration in rats, BBR is absorptive by gastrointestinal system, involving the reciprocal transformation between BBR and dihydroberberine (Feng et al., 2015). BBR has wildly distribution in the liver, kidney, muscle, lung, brain and heart (decreasing order) (Tan et al., 2013). In liver, BBR metabolizes in five ways, reduction, demethylation, demethylenation, hydroxylation and subsequent conjugation reactions (Wang et al., 2017b). Cytochrome P450 (CYP450) isoenzymes 2D6, 1A2 and 3A4 (Li et al., 2011) play an important role in metabolism and jatrorrhizine, thalifendine, berberrubine and demethylene-berberine are the main products of phase I metabolism. Meanwhile, the four metabolites can be detected in feces, urine and bile (Ma et al., 2013). The main way of metabolism of BBR and its metabolites is through feces.

BBR has poor intestinal absorption and oral bioavailability in humans and rats. Its absolute oral bioavailability is only 0.37 ± 0.11% (Feng et al., 2020). In humans, after oral administrations of 400 mg BBR, the mean maximum plasma concentration is approximately 0.4 ng/ml (Hua et al., 2007). The most ionized form of BBR under physiological conditions (pKa = ∼15) results in lower oral bioavailability (Spinozzi et al., 2014). P-glycoprotein (P-gp) expressed in the apical membrane of intestinal mucosal cells secretes xenobiotics into the lumen, thereby limiting the net intestinal absorption of BBR (Maeng et al., 2002). In addition, extensive intestinal first-pass elimination leads to BBR remaining in the gastrointestinal lumen, which is finally excreted in the stool (Liu et al., 2010; Wang et al., 2017c). Moreover, after absorption through the intestine, BBR is widely distributed in organs, predominantly in liver (Liu et al., 2010; Tan et al., 2013), thereby making the BBR content of the organs much higher than the plasma concentration (Wang et al., 2017b). *In vivo*, BBR undergoes extensive metabolism and simultaneously exists with its metabolites (Wang et al., 2017b) (Figure 3).

The acute toxicity of BBR depends on the method of administration, and basic acute toxicity studies have illustrated that the median lethal dose (LD_50_) of BBR from intravenous and intraperitoneal injection is 9.0386 and 57.6103 mg/kg, respectively, in mice. Interestingly, no LD_50_ was found after oral administration, possibly because of its poor oral bioavailability (Kheir et al., 2010). Previous studies have revealed that BBR is cardiotoxic, leading to total amplitude and beating rate inhibition at 10 μM in cardiomyocytes (Zhang et al., 2018). Animal experiments have suggested that BBR exerts toxic and teratogenic effects in developing zebrafish embryos at 400 mg/L for a-48 h administration, as evidenced by pericardial edema, damaged heart morphogenesis, vascular defects, and death (Martini et al., 2020). BBR treatment during vertebrate development leads to the impairment of cardiovascular system morphogenesis and functionality, suggesting it should be used with caution during pregnancy and lactation. In addition, BBR shows a risk of potential neurotoxicity and immunotoxicity (Kysenius et al., 2014; Mahmoudi et al., 2016), and high doses of BBR (10 mg/kg) can suppress both cellular and humoral immune functions in treated hosts. BBR at 5 mg/kg appeared to affect only delayed-type hypersensitivity responses and lymphoproliferation (Mahmoudi et al., 2016). Moreover, several studies have shown that the concomitant use of BBR with macrolides or 5-aminosalicylic acid may induce cardiac or splenic toxicity, respectively (Zhi et al., 2015; Li et al., 2016). However, the above-mentioned data are derived from animal experiments, and recent clinical trials have shown that BBR is well tolerated in the human body with only some gastrointestinal side effects (Zhang et al., 2020c; Zhao et al., 2021b). Cardioprotective effects have also been confirmed (Qing et al., 2018; Mercurio et al., 2020; Zhao et al., 2021a). These differences may be related to administration time, concentration, and species. Overall, current research proves that BBR is safe for humans, but the rare embryo- and immune-related toxicity suggests its potential risk for pregnant women and children. In addition, the interaction between BBR and drugs requires further research (Table 2).

**TABLE 2:** Toxicity researches of BBR.

Animals/cell lines	Dose	Administration method	Duration	Detail	Ref.
Fourth instar larvae	10 mg/L BBR plus near UVA	—	24 h	Decreased larval survival	Philogène et al. (1984)
Wistar and Sprague-Dawley rats	2, 10, 20 μg/g	intraperitoneal injection	7 days	Higher mean serum bilirubin concentration	Chan (1993)
Balb/c mice	10 mg/kg	intraperitoneal injection	14 days	Cellular and humoral immune functions suppression	Mahmoudi et al. (2016)
	5 mg/kg			Delayed-type hypersensitivity responses, lymphoproliferation	
Zebrafish embryos	100 mg/L	—	24–96 h	Teratogenic effect, developmental toxicity, pericardial edema, cardiac looping defects, late heart morphogenesis impairment, early cardiac functionality impairment	Martini et al. (2020)
Cerebellar granule neurons, hippocampal neurons	0.01–10 µM		0.5–24 h	Severe disruption of neuritic and nuclear integrity, functional and morphological alterations of neuronal mitochondria	Kysenius et al. (2014)
Sprague-Dawley rat cardiomyocytes	10 μM	—	24 h	Cardiac arrest, total beating rate and amplitude inhibition	Zhang et al. (2018)

Note- :no relevant data.

## Mechanisms of BBR Anticancer Effects

BBR is shown to exert pro-apoptotic effects in multiple cancers (Table 3). In the following section, we provide a comprehensive summary of the anticancer mechanisms.

**TABLE 3:** Pro-apoptotic effect of BBR on the three cancers *in vitro*.

Disease	Cell lines	Dose of BBR	Duration	Detail	Ref.
Breast cancer	MCF-7 cells	1, 10 μM	24, 48, 72 h	Cell proliferation inhibition, cell cycle arrest	Sakaguchi et al. (2020)
		100 μM	72 h	Cell death	
	MCF-7 cells, MDA-MB-231 cells	50, 100 μM	24, 48 h	Apoptosis	Sun et al. (2019)
	MDA-MB-468 cells	3, 6, 12 μM	1, 2, 3, 4 days	Cell proliferation inhibition	Lin et al. (2019)
	MDA-MB-231 cells	6.25, 12.5, 25 μM			
	MDA-MB-453 cells	2.5, 5, 10 μM			
	BT-549 cells	5, 10, and 20 μM			
	HCC70 cells	0.5, 1 µM	120, 144 h	Apoptosis	El Khalki et al. (2020)
	BT-20 cells				
	MDA-MB-468 cells				
	MDA-MB-231 cells	5 μg/ml	48 h	Apoptosis	Zhao et al. (2017)
	MDA-MB-231 cells	2.5–100 μg/ml	48 h	Cell proliferation, growth and metastasis inhibition	Yao et al. (2019)
	MDA-MB-231 cells	25 μmol/L	48 h	Cell migration inhibition	Zhao and Zhang. (2020)
	ZR-75-30 cells	0.78, 1.56, 3.12 µM	48 h	Cell proliferation, migration inhibition	Ma et al. (2017)
	MDA-MB-231 cells	20, 40, 80, 160 μmol/L	48, 72 h	DNA breaks	Gao et al. (2019)
	Multidrug-resistant MCF-7 cells	80, 160, 320 µM	24, 48, 72 h	Apoptosis	Pan et al. (2017)
	MCF-7 cells, MDA-MB-231 cells	100 µM	48,72 h	Apoptosis	Jabbarzadeh Kaboli et al. (2019)
	MCF-7 cells, MDA-MB-231 cells	20, 40, 80 µM	24, 48 h	Cell cycle arrest	Tak et al. (2019)
	Dox resistant MCF-7 cells	50, 100, 150, 200 µM	48 h	Cell growth inhibition	Wang et al. (2020)
Lung cancer	H1975 cells, A549 cells	6.25, 12.5, 25, 50 and 100 μM	24, 48, 72 h	Cell growth, migration and invasion inhibition, cell cycle arrest	Zheng et al. (2018a)
	H1299 cells, A549 cells	20, 100 μM	48 h	Apoptosis	Fu et al. (2013)
	A549 cells	3.15, 6.25, 12.5, 25 μM	48 h	Apoptosis	Kumar et al. (2020)
		40, 80,120 μM	48 h	Apoptosis	Chen et al. (2019)
	H1975 cells, H1650 cells	0.78125–12.5 μM	24 h	Cell growth inhibition	Fan et al. (2018)
	A549 cells	20–200 μM	24, 36, and 48 h.	Apoptosis	Kalaiarasi et al. (2016)
	H1975 cells	25 μmol/L	24 h	Apoptosis	Zheng et al. (2020)
Liver cancer	HepG2 cells	5 μg/ml	4 days	Limits DOX-exacerbated HCC repopulation	Zhang et al. (2019b)
	Hep3B cells, BEL-7404 cells	50, 75, 100, 125 μM	12, 24, 48 h	Cell proliferation inhibition	Zhang et al. (2019c)
	HepG2 cells	10, 50, and 100 μg/ml	24, 48, 72 h	Cell proliferation inhibition, apoptosis	Li et al. (2017)
	HepG2 cells	50, 100 μM	18, 24 h	Apoptosis	Saxena et al. (2018)
	Huh-7.HCVrep cells	100 μM	24, 48 h	Apoptosis	Tai et al. (2020)
	MHCC97L Cells, PLC/PRF/5 cells	7.8125–1,000 μM	24 h	Apoptosis	Guo et al. (2020)
	HepG2 cells, Hep3B cells, SNU-182 cells	10, 20, 50, 100 µM	72 h	Cell proliferation inhibition	Chuang et al. (2017)
	HepG2 cells	100 μM	24 h	Cell growth inhibition	Chen et al. (2016)
	HepG2 cells	50, 100 μM	6 h	Cell invasion and metastasis inhibition	Wang et al. (2016)
	HepG2 cells	3.125 μM	7 days	Chemotherapy-induced apoptosis promoted migration inhibition	Zhao et al. (2020)
	Huh-7 cells, HepG2 cells	30 μM	14 days	Cell cycle arrest	Li et al. (2018a)
	Huh7 cells	50 μM	72 h	Apoptosis	Vishnoi et al. (2021)
	MHCC97-H cells, HepG2 cells	50, 100, 200 μM	24 h	Apoptosis	Song et al. (2019b)

Note: Huh-7.HCVrep cells: AB12-A2 Huh-7 replicon cells carrying the HCV genotype 1b subgenomic RNA.

### Breast Cancer

**FIGURE 4:**
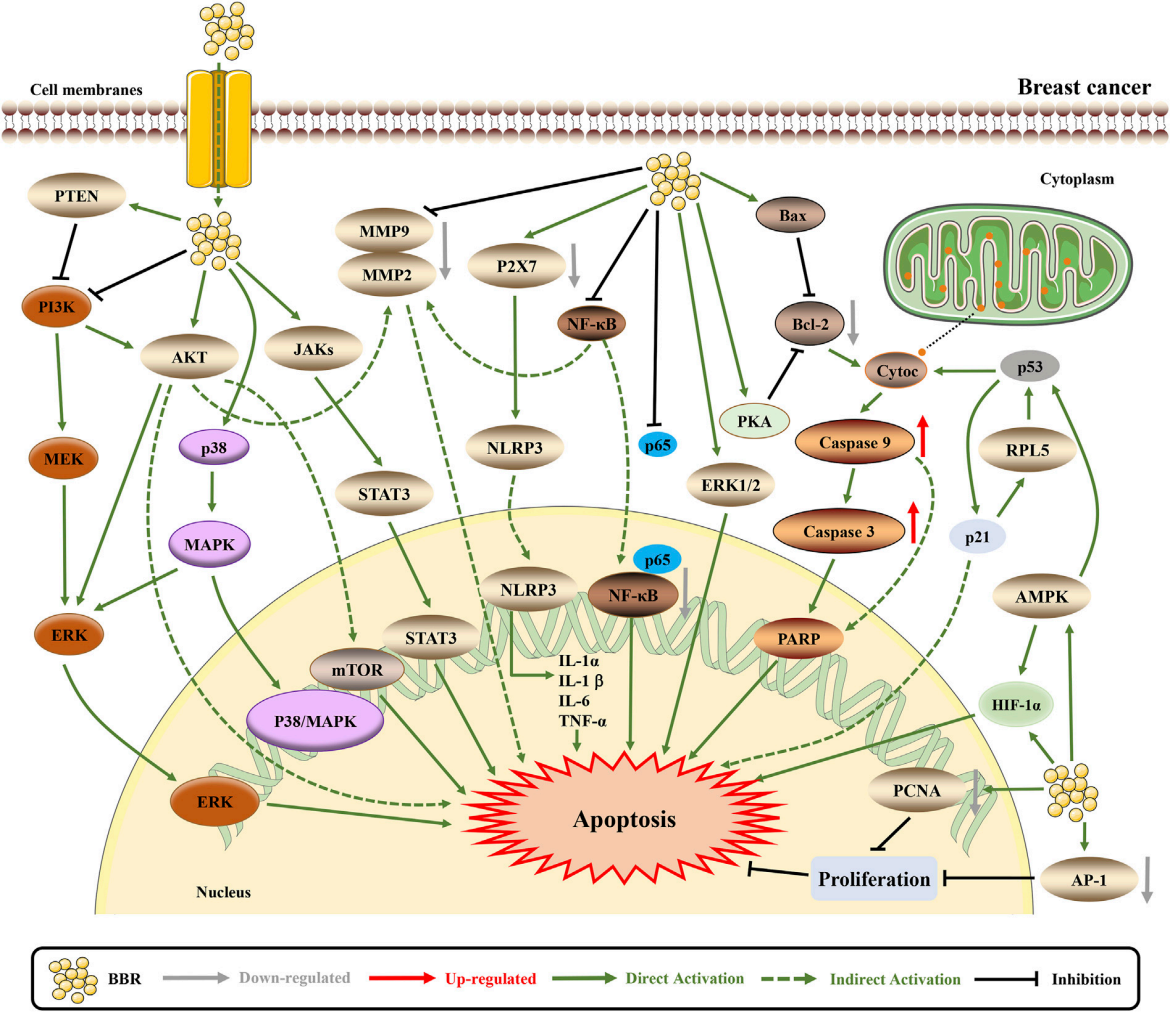
The pro-apoptotic mechanisms of BBR in breast cancer. BBR exerts anti-cancer effects by influencing breast cancer related pathways. BBR induces apoptosis through downregulating MTDH (Sun et al., 2019). BBR actives the mitochondrial apoptotic pathway and upregulates AMPK to promote apoptosis (Pan et al., 2017; Zhao et al., 2017; Ma et al., 2020). Furthermore, BBR downregulates AKT/ERK pathway to induce cell cycle arrest and apoptosis (Lin et al., 2019). BBR inhibits NLRP3 and NF-κB inflammasome pathway to suppress inflammation and EMT, thus inducing apoptosis (Karnam et al., 2017; Yao et al., 2019; Zhao and Zhang, 2020). The mechanism of BBR inhibiting cell proliferation, invasion and growth may be induced by EGFR/ERK and EGFR/AKT signaling pathways (Kim et al., 2018c; Jabbarzadeh Kaboli et al., 2019). BBR affects certain protein expression and structure via VASP, and downregulates AP-1 and PCNA to inhibit cell proliferation and migration, which also decreases resistance to apoptosis (Su et al., 2016; Karnam et al., 2017; Jeong et al., 2018).

Numerous experiments have shown that BBR is effective for treating breast cancer through diverse mechanisms, including the inhibition of cancer cell proliferation, metastasis; regulation of metabolism, the immune system, and cancer-related pathways; and the promotion of DNA damage (Figure 4).

### BBR Inhibits Cell Proliferation to Induce Apoptosis

Proliferation and apoptosis are considered opposing cellular phenomena, and the occurrence of tumors is believed to be related to blocked apoptosis and abnormal proliferation. According to the literature, higher doses of BBR (100 μM) are predominantly distributed in the nucleolus, which causes a nucleolar stress response to upregulate p53 and induce cell proliferation inhibition and cell death (Sakaguchi et al., 2020). In addition, BBR downregulates metadherin (MTDH) to exert an antiproliferative effect (Sun et al., 2019). In triple-negative breast cancer (TNBC) cell lines, BBR inhibits the proliferation of TNBC cells with IC_50_ values ranging from 0.19 to 16.7 µM including different pathways, such as AKT/extracellular regulated protein kinases (ERK) pathways and p38. Moreover, the cell cycle kinase complex cyclin A/cyclin-dependent kinase (CDK) 1, and cyclin D/CDK4 are suppressed (Lin et al., 2019; El Khalki et al., 2020). Furthermore, recent studies have indicated that, with cell proliferation inhibition, BBR induces apoptotic cell death through the activation of caspase-9/cytochrome c; the cleavage of poly ADP-ribose polymerase (PARP), caspase-7, and caspase-8 proteins, and upregulation of pro-apoptotic B-cell lymphoma-2 (Bcl-2) proteins via p53 (Zhao et al., 2017; Carneiro and El-Deiry, 2020; El Khalki et al., 2020).

### BBR Inhibits Cell Invasion and Metastasis to Potentially Induce Apoptosis

Metastasis is a characteristic of tumor invasiveness. Several studies have shown that BBR inhibits colony formation and migration by decreasing the phosphorylation of c-Jun and c-Fos (Zhao et al., 2017; Yao et al., 2019; Zhao and Zhang, 2020). Recent studies have indicated that BBR downregulates ephrin-B2 and transforming growth factor beta 1 (TGF-β1) to decrease the expression levels of matrix metalloproteinase (MMP)2 and MMP9, thereby inhibiting cell growth and migration (Ma et al., 2017; Kim et al., 2018a). MMPs are known to be involved in all steps of tumor progression. For example, MMP11 inhibits apoptosis and promotes cancer development. As an MMP inhibitor, BBR may be able to regulate MMPs and tissue inhibitors of MMPs to induce apoptosis (Cabral-Pacheco et al., 2020). In addition, BBR suppresses the toll-like receptor 9 (TLR9)-myeloid differentiation factor 88 (MyD88)–nuclear factor kappa-B (NF-κB) pathway and partly reverses doxorubicin (Dox)-exacerbated breast cancer metastasis (Zheng et al., 2021). NF-κB is a transcription factor that reduces apoptosis and has been shown to be involved in cell apoptosis in the phosphoinositide-3-kinase (PI3K)/AKT/NF-κB/MMP9 signaling pathway (Yang et al., 2019). The overexpression of vasodilator-stimulated phosphoprotein (VASP) and fibronectin (FN) is related to the poor prognosis of breast cancer, BBR not only binds to VASP to induce changes in its secondary structure but also decreases the expression of FN to inhibit cell proliferation and migration (Su et al., 2016; Jeong et al., 2018). In particular, serine phosphorylation of VASP regulates colon cancer cell survival and apoptosis, while FN protects lung cancer cells against docetaxel-induced apoptosis (Ali et al., 2015; Qin et al., 2016). Ultimately, the inhibition of cell invasion and metastasis may be a potential target for BBR to induce apoptosis.

### BBR Regulates Metabolism to Synergistically Induce Apoptosis

Metabolic disorders are characterized by several pathological states such as chronic inflammation, dyslipidemia and insulin resistance, which are related to the incidence of breast cancer. Therefore, targeting the metabolism to treat cancer is a feasible approach. BBR has the ability to regulate metabolism or metabolic functions to affect the outcome of breast cancer (Cazzaniga and Bonanni, 2018). Previous studies have shown that BBR interferes with breast cancer cell metabolism and induces apoptosis via a mitochondrial-dependent pathway (Patil et al., 2010; Tan et al., 2015a; Tan et al., 2015b). As mentioned earlier, BBR targets p53, which regulates multiple metabolic pathways, including glucose, lipid, mitochondrial, serine, nucleotide metabolism and oxidative phosphorylation, thereby synergistically inducing apoptosis (Liu et al., 2019a). Although its specific mechanism remains unclear, the use of BBR in breast cancer patients has been recommended (Cazzaniga and Bonanni, 2018).

### BBR Regulates the Immune System to Active Apoptosis Pathway

Cancer immunosurveillance usually challenges cells that have undergone tumor transformation. Only the most immune-evasive or highly mutagenic tumor cells can escape immune surveillance and produce clinically relevant tumors. Therefore, cancer cells in established tumors can resist antitumor immunity. Thus, overcoming the immune-evasive phenotype has become a new strategy for effective cancer therapies (Garg and Agostinis, 2017). Physiologically, the immune system can activate the mitochondrial apoptosis pathway and the Fas death receptor apoptosis pathway without causing inflammation or tissue destruction (Nagata and Tanaka, 2017). Recently, BBR and exercise have been shown to slow the progression of breast cancer in 4T1 tumor-bearing rats. Synergistic therapy regulates intestinal microbial metabolites to improve the immune system by activating the mitochondrial apoptosis pathway and the Fas death receptor apoptosis pathway, thereby exerting anticancer effects (Ma et al., 2020). Collectively, these results provide evidence that BBR regulates immunity during cancer treatment.

### BBR Promotes DNA Damage to Induce Apoptosis

DNA fragmentation is a hallmark of apoptosis, and BBR can induce DNA fragmentation in MDA-MB-231 cells. Further studies have shown that BBR markedly downregulates the levels of X-ray cross-complementing protein 1 and excision repair cross-complementing group 1, thereby inhibiting cell DNA repair and sensitizing MDA-MB-231 cells to chemotherapeutic drugs (Gao et al., 2019). Another study showed (El Khalki et al., 2020) that after 120 h of BBR treatment, both BT-20 and HCC70 showed significant H2A.X variant histone (H2AX) phosphorylation, which is a sign of DNA damage. Finally, DNA damage promotes p53 expression, leading to the activation of apoptosis (Hafner et al., 2019).

### BBR Suppresses Inflammation to Induce Apoptosis

Infection and inflammation account for approximately a quarter of the causes of cancers, and tumors can be described as wounds that never heal and are infiltrated by numerous inflammatory and immune cells. Tumor-related chronic inflammation is generally believed to be a sign of cancer that promotes the progression to metastatic stage, and plays an important role in tumor development and treatment (Suarez-Carmona et al., 2017; Murata, 2018). After BBR treatment, P2X7-mediated NLR family pyrin domain containing 3 (NLRP3) inflammasome activation was inhibited, as evidenced by the suppressed expressing of NLRP3, caspase-1, and interleukin 1 beta (IL-1β) in MDA-MB-231 cells (Yao et al., 2019). BBR can also inhibit the secretion of tumor necrosis factor alpha (TNF-α) and interleukin-6 (IL-6) by increasing the expression of the nuclear factor of kappa light polypeptide gene enhancer in B-cells inhibitor, alpha (IκBα), thus inhibiting p65 protein and NF-κB (Zhao and Zhang, 2020). Similarly, in breast cancer-bearing rats treated with BBR (50 mg/kg), the increased levels of lipid peroxide (malonaldehyde), pro-inflammatory cytokines (IL-1β, IL-6 and TNF-α), enzymatic antioxidants (superoxide dismutase and catalase), non-enzymatic antioxidants (glutathione and vitamin C), and transcription factor NF-κB were significantly decreased by the administration of BBR. Furthermore, NF-κB and proliferating cell nuclear antigen (PCNA) in breast cancer were also downregulated (Karnam et al., 2017). Finally, with NF-κB downregulation, apoptosis was induced in breast cancer cells.

### BBR Regulates Cancer-Related Pathways to Induce Apoptosis

#### AMPK Pathway

In breast cancer, AMPK phosphorylation induces multiple cellular responses, including cell growth inhibition, cell cycle arrest and apoptosis (Ponnusamy et al., 2020b). Salt-inducible kinase 3 (SIK3) belongs to the AMPK-related kinase family. The combination of emodin and BBR attenuates SIK3, leading to cell cycle arrest, decreased cell growth and increased apoptosis in breast cancer cells (Ponnusamy et al., 2020a). Interestingly, low-dose BBR enhances Dox sensitivity to drug-resistant breast cancer through the AMPK- hypoxia-inducible factor-1α (HIF-1α) -P-gp pathway. High-dose BBR directly induces apoptosis by activating the AMPK-p53 pathway. Therefore, BBR is both a chemotherapy sensitizer and chemotherapy drug (Pan et al., 2017).

#### MAPK Pathway

Overwhelming evidence indicates that the aberrant activation of the MAPK pathway contributes to breast cancer growth, invasion, metastasis, and reduced apoptosis. For example, MAPK promotes breast cancer cell invasion and metastasis via interleukin 32 (Wen et al., 2019). MAPK promotes breast cancer cell growth via linc-RoR (Peng et al., 2017). Downregulated MAPK has been shown to induce apoptosis and inhibit the proliferation of breast cancer cells (Zhu et al., 2020). The epidermal growth factor receptor (EGFR) is a surface marker of breast cancer. Dysregulation of EGFR/MAPK is common in this disease. BBR downregulates EGFR/MAPK to induce apoptosis (Jabbarzadeh Kaboli et al., 2019). BBR also reduces the expression of interleukin-8 (IL-8) by downregulating the EGFR/mitogen-activated protein kinase (MEK)/ERK pathway to induce cell growth and inhibit invasion (Kim et al., 2018c).

#### AKT Pathway

Aberrant AKT expression is associated with increased invasion, metastasis and drug resistance (Shariati and Meric-Bernstam, 2019). Several lines of evidence have suggested that BBR induces cell cycle arrest and death by suppressing AKT (Tak et al., 2019; Ponnusamy et al., 2020a). BBR modulates the phosphatase and tensin homolog (PTEN)/AKT/mammalian target of rapamycin (mTOR) signaling pathway to reverse Dox resistance and induce apoptosis (Wang et al., 2020).

### BBR Reverses Drug Resistance to Induce Apoptosis

Lapatinib is a new type of human epidermal growth factor receptor 2 (HER2)/EGFR tyrosine kinase inhibitor used for the treatment of HER2-positive breast cancer. However, acquired drug resistance is inevitable. Studies have shown that BBR can increase the level of reactive oxygen species (ROS) to induce apoptosis in lapatinib-resistant cells and reverse the drug resistance of breast cancer cells (Zhang et al., 2016). In addition, BBR and Dox synergistically enhanced the inhibitory effect on cancer cells by reducing P-gp and multidrug resistance protein 1 expression with the best BBR/Dox ratio of = 2:1 (Qian et al., 2021).

### Lung Cancer

**FIGURE 5:**
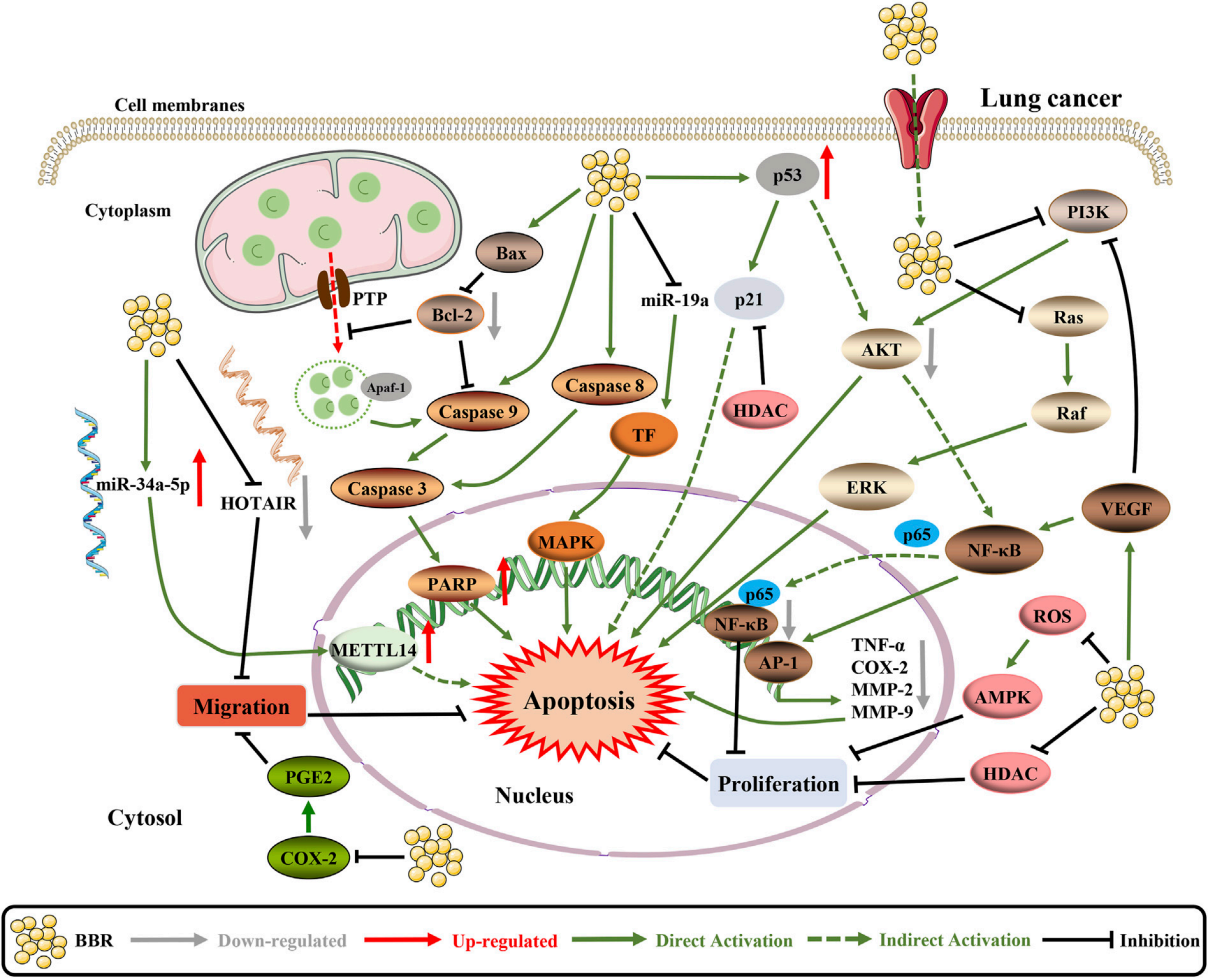
The pro-apoptotic mechanisms of BBR in lung cancer. BBR increases the activity of the Bcl-2/Bax signaling pathway and inhibits the VEGF/NF-κB/AP-1 signaling pathway to enhance cell apoptosis (Li et al., 2018b). BBR upregulates tumor suppressor genes (p21 and p53) mRNA and protein by inhibiting HDAC (Kalaiarasi et al., 2016). On the top of this, BBR can regulate the expression and interaction of HOTAIR and miR-34a-5p in human lung cancer cells, which significantly inhibits EMT and induces apoptosis (Zheng et al., 2020). In addition, BBR inhibits the cell proliferation by targeting PI3K/AKT and ROS/AMPK signaling pathways to activate apoptosis (Fu et al., 2013; Bhattacharyya et al., 2017).

As documented in the literature, BBR exhibits anti-lung cancer activity via multiple molecular mechanisms (Figure 5). BBR can significantly inhibit the growth of lung cancer cells by reducing the protein expression of transcription factors Sp1 and 3-phosphoinositide dependent protein kinase 1 (PDPK1), both of which are related to cancer growth and progression (Zheng et al., 2018a). Furthermore, BBR induces apoptosis by modulating cell cyclins, inflammation, and the PI3K/AKT pathway. However, it was also observed that the proliferation inhibitory effect of BBR was transient, and no significant inhibitory effect was observed in the second-generation cells (Fu et al., 2013; Kumar et al., 2020). Another study showed that the tissue factor (TF) is a direct inhibitory target of miR-19a in non-small cell lung cancer (NSCLC) cells and BBR induces apoptosis through the miR-19a/TF/MAPK axis (Chen et al., 2019). In addition, BBR can cause mitochondrial dysfunction. By activating the ROS/AMPK pathway, BBR inhibits lipogenesis and cell proliferation (Fan et al., 2018). The combination of BBR and UVA can significantly increase cytotoxicity, and the phototoxicity of BBR is mediated by the production of ROS, mitochondrial membrane permeabilization and caspase-9/caspase-3 activation (Bhattacharyya et al., 2017). BBR also mediates epigenetic reprogramming by inhibiting histone deacetylase (HDAC) to downregulate oncogenes such as TNF-α, cyclooxygenase (COX)-2, MMP2, and MMP9 and cancer suppressor genes (p21 and p53) mRNA and protein upregulation. Besides, BBR regulates Bcl-2/Bcl-2 associated X protein (Bax) family proteins and triggers the caspase cascade apoptotic pathway (Kalaiarasi et al., 2016).

Epithelial-mesenchymal transition (EMT) is a process through which epithelial cells acquire mesenchymal features. EMT is associated with cancer initiation, invasion, metastasis, and resistance to therapy (Nieto et al., 2016). BBR and gefitinib can regulate the expression and interaction of HOX transcript antisense intergenic RNA (HOTAIR) and miR-34a-5p in human lung cancer cells to inhibit EMT. HOTAIR is an important oncogenic lncRNA, that is involved in tumorigenesis and invasion, and miR-34a-5p is a tumor suppressor. HOTAIR is significantly elevated in NSCLC cells and promotes migration and invasion, whereas depletion of HOTAIR reduces the migration and invasion ability of NSCLC cells (Zheng et al., 2020).

*In vitro* studies have shown that continuous Dox treatment causes transformation of HL-60 cells to N2 neutrophils, thereby inducing chemotherapy resistance. The combined treatment of Dox and 2 µM BBR caused HL-60 cells to differentiate into N1 neutrophils and stimulates immune clearance in HL-60 cells. In addition, BBR can also downregulate the expression of Dox-derived neutrophils CD133 and CD309 to prevent chemotherapy sensitivity and immune rejection (Zhang et al., 2019d).

### Liver Cancer

**FIGURE 6:**
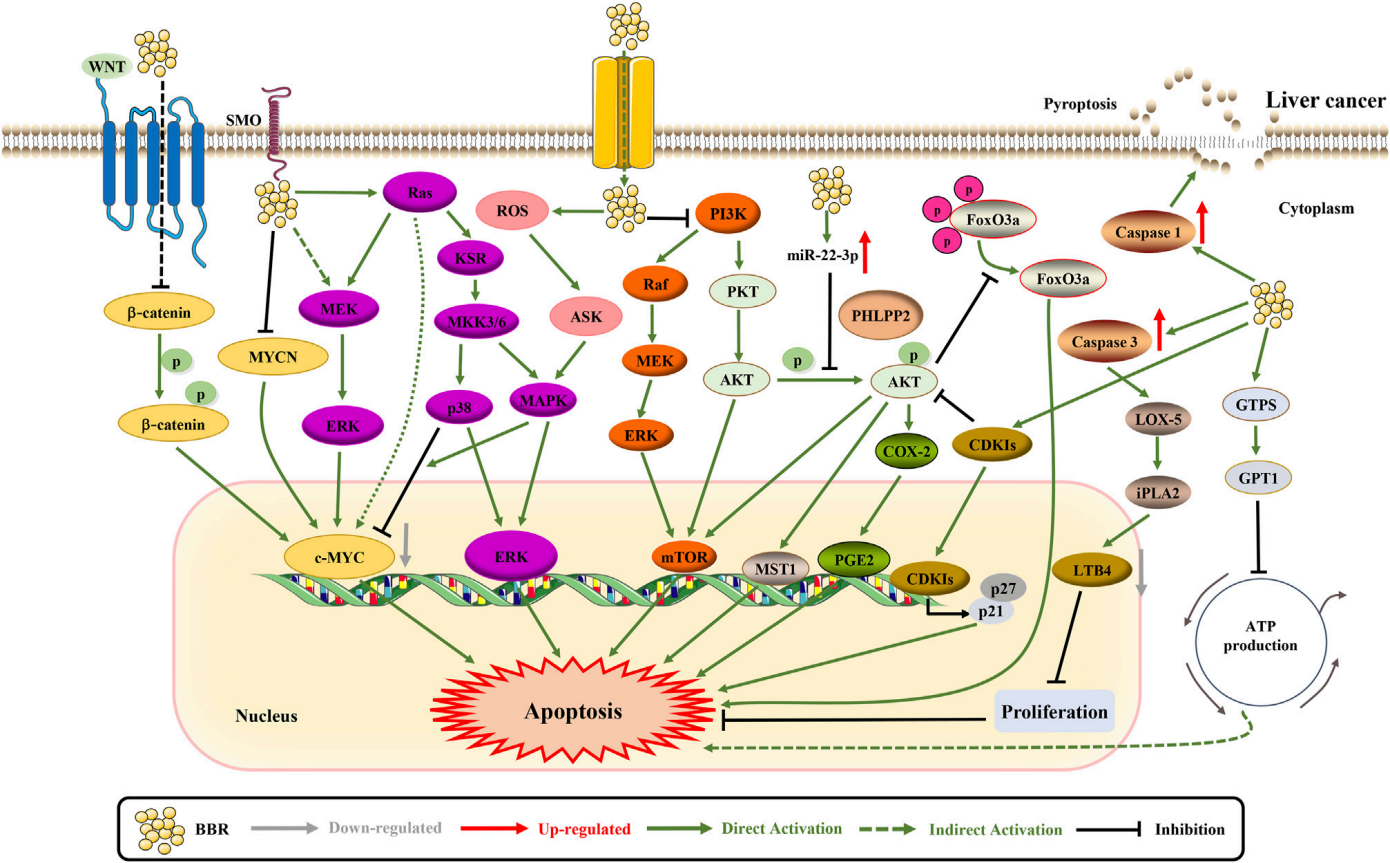
The pro-apoptotic mechanisms of BBR in liver cancer. BBR promotes apoptosis by suppressing the expression of SLC1A5 and c-MYC (Zhang et al., 2019c; Tai et al., 2020). BBR increases the expression of CDKIs p21^Cip1^ and p27^Kip1^ via regulating the AKT/FoxO3a signaling pathway (Guo et al., 2020). Likewise, BBR induces apoptosis through iPLA2/LOX-5/LTB4 pathway and PHLPP2-AKT-MST1 axis (Saxena et al., 2018; Zhao et al., 2020). Besides, BBR inhibits cell proliferation through ERK1/2 and GPT1 axis (Chuang et al., 2017; Guo et al., 2020). BBR can regulate adenylate cyclase signaling in association with MKK3/6 stabilization to exert necroptosis (Sengupta et al., 2017). BBR upregulates miR-22-3p to inhibit cell proliferation (Chen et al., 2016). Furthermore, BBR simultaneously inhibits NF-κB p65 and β-catenin translation to promote apoptosis (Li et al., 2017). The apoptosis promotion of BBR may also be related to the inhibition of PI3K/AKT, p38 MAPK/ERK-COX2 and HIF-1α (Luo et al., 2019b; Song et al., 2019b; Qi and Liu, 2021).

Through continued research, numerous anti-liver cancer activities of BBR are being recognized (Figure 6).

### BBR Inhibits Cell Proliferation and Growth to Induce Apoptosis

Cancer cells undergo specific metabolic reprogramming to sustain their proliferation. In Hep3B and BEL-7404 cells, BBR treatment decreases the rate of glutamine uptake by inhibiting solute carrier family 1 member 5 (SLC1A5) to in turn inhibit cell proliferation (Zhang et al., 2019c). In addition, BBR treatment inhibits chemotherapy-exacerbated hepatocellular carcinoma (HCC) cell population through the caspase-3-calcium-independent phospholipase A2 (iPLA2) -COX-2 pathway (Zhang et al., 2019b). BBR treatment can also inhibit cell proliferation by targeting Sp1 to upregulate miR-22-3p and suppress NF-κB p65 to induce apoptosis (Li et al., 2017). The pleckstrin homology domain leucine-rich repeat protein phosphatase 2 (PHLPP2), a tumor suppressor gene, can upregulate PHLPP2 from the PHLPP2-AKT-mammalian sterile 20-like kinase 1 (MST1) axis, exerting anti-proliferative effects (Saxena et al., 2018). A recent study revealed that BBR treatment enhanced ROS production and increased apoptosis (Tai et al., 2020). BBR treatment inhibits the growth of HCC cells by inhibiting glutamic-pyruvic transaminase 1 (GPT1) (Guo et al., 2020). Similarly, BBR treatment inhibits HCC cell proliferation by activating ERK1/2 phosphorylation (Chuang et al., 2017). The combination of S-allyl-cysteine and BBR can regulate adenylate cyclase signaling in association with stabilization of mitogen-activated protein kinase 3/6 (MKK3/6) to inhibit necroptosis and proliferation (Sengupta et al., 2017). BBR also regulates miR-22-3p to inhibit HCC development and progression (Chen et al., 2016). Overall, these results suggest that BBR induces apoptosis by inhibiting cell proliferation and growth.

### BBR Inhibits Cell Invasion and Metastasis to Potentially Induce Apoptosis

Cyclin D1 is overexpressed in HCC and is associated with invasiveness. Studies have shown that BBR can inhibit cyclin D1 expression in liver cancer cells (Wang et al., 2016). Apoptotic cancer cells induced by chemotherapeutics can also change the tumor microenvironment by activating the lipoxygenase (LOX) pathway, and release inflammatory factors such as leukotriene B4 (LTB4) to stimulate the adhesion and migration of a small number of surviving cells. BBR can reverse FN adhesion and migration in HepG2 cells by inhibiting the iPLA2/LOX-5/LTB4 pathway (Zhao et al., 2020).

### BBR Regulates Cancer-Related Pathways to Induce Apoptosis

First, BBR promotes the expression of cyclin-dependent kinase inhibitors (CDKIs) p21^Cip1^ and p27^Kip1^ by regulating the AKT/Forkhead box O3a (FoxO3a)/S-phase kinase-associated protein 2 (Skp2) axis and further induces HCC cell cycle arrest (Li et al., 2018a). Second, BBR antagonizes the β-catenin pathway to induce apoptosis, thereby reducing the survival rate of HCC cells (Vishnoi et al., 2021). However, BBR combined with HMQ1611 has also been shown to inhibit the WNT/β-catenin pathway and inhibit the proliferation and metastasis of HCC (Dai et al., 2019). Third, BBR can inhibit the PI3K/AKT pathway to inhibit cell growth, migration and invasion, and induce apoptosis (Song et al., 2019b). In Western countries, liver cancer is increasingly caused by nonalcoholic fatty liver disease/nonalcoholic steatohepatitis (NAFLD/NASH). Interestingly, BBR might alleviate NASH-HCC via the p38MAPK and ERK/COX-2 pathways (Luo et al., 2019b).

### BBR Induces Cell Pyroptosis

Pyroptosis is a caspase-1-dependent programmed cell death. However, the role of pyroptosis in HCC remains unclear. Pyrolysis in HCC tissues and cells is suppressed. Administration of BBR inhibited the viability, migration and invasion of HepG2 cells by inducing cell pyrolysis, which was attenuated by the caspase-1 inhibitor Ac-YVAD-CMK. In summary, pyroptosis is involved in the pathogenesis of HCC and may be a new target for HCC treatment (Chu et al., 2016).

### BBR Promotes Chemosensitivity and Radiosensitivity to Induce Apoptosis

Regorafenib resistance is an important barrier to the treatment of advanced liver cancer. BBR enhances the cytotoxicity of regorafenib in liver cancer cells. The combination of these two factors significantly inhibits the proliferation of HCC cells and induces apoptosis (Wang et al., 2021). Similarly, BBR can induce cell apoptosis and inhibit proliferation by resisting sorafenib resistance (Huang et al., 2018). 10-hydroxycamptothecin (10-HCPT) is an effective topoisomerase I inhibitor used in the treatment of liver cancer. The high expression of HIF-1α in liver cancer tissues is related to 10-HCPT resistance, which is considered a potential cancer target for natural products (Li et al., 2020). The upregulation of HIF-1α has been confirmed to exert anti-apoptotic effects (Tang et al., 2021). Conversely, BBR can inhibit the expression of HIF-1α as a synergistic treatment for liver cancer (Qi and Liu, 2021). Radiotherapy, which can be enhanced by BBR, is a common treatment for liver cancer. For instance, BBR enhances radiation-induced oxidative stress and apoptosis in Huh7 and HepG2 cells by suppressing the nuclear factor erythroid 2–related factor 2 (Nrf2) signaling pathway (You et al., 2019).

## Anticancer Effect of BBR Against Other Cancers

There is growing evidence that BBR exhibits other anticancer activities. Nasopharyngeal carcinoma (NPC) is a malignancy derived from the epithelial cells of the nasopharynx cavity, and is closely associated with Epstein-Barr virus infection, BBR decreases the expression of EBNA1 to exhibit an antitumor effect against NPC (Wang et al., 2017a). BBR represses human gastric cancer cell growth by inducing cytostatic autophagy via the inhibition of MAPK/mTOR/p70 ribosomal S6 protein kinase and AKT (Zhang et al., 2020a). Moreover, BBR can regulate metabolism and exert anticancer effects. Lipid metabolism is a significant component of energy homeostasis, and BBR may partially inhibit colon cancer growth by targeting the SREBP cleavage-activating protein/sterol regulatory element-binding protein-1 pathway driving lipogenesis (Liu et al., 2020b). BBR also inhibits overactive glucose metabolism in colon cancer cells by suppressing mTOR-dependent HIF-1α protein synthesis (Mao et al., 2018). Furthermore, BBR promotes the degradation of sonic hedgehog mRNA in colorectal cancer cells, interrupting the paracrine hedgehog signaling pathway activity thus suppressing colorectal cancer growth (Shen et al., 2021). Additionally, BBR regulates the TGF-β pathway and reverses EMT in normal colonic epithelial cells (Huang et al., 2019a). In gynecology, BBR has been shown to suppress the growth and metastasis of endometrial cancer cells via miR-101/COX-2, and the combination therapy of BBR and cisplatin markedly enhances ovarian cancer cell death by inducing apoptosis and necroptosis, which may improve the anticancer effect of chemotherapy drugs (Liu et al., 2019b). Collectively, these results indicate that BBR has a wide range of anticancer effects.

## Anticancer Effect of BBR Derivatives

BBR derivatives have better bioavailability, specific targeting, and stronger anticancer effects than those of BBR. The BBR derivative 8-cetylBBR (HBBR) is more effective for targeting lungs, and its anticancer properties are also significantly stronger than those of BBR. Animal experiments have proven that the oral administration of HBBR at a dose of 10 mg/kg significantly inhibited tumor growth and was more effective than the 120 mg/kg dose of BBR treatment (Xiao et al., 2018). Esters, amides, and sulfonates of BBR have also been developed for small-molecule cancer immunotherapy, such as 2,3-methylenedioxy-9-((2,2,3,3-tetramethylcyclopropane-1-carbonyl) oxy)-10-methoxyprotoberberine chloride and 2,3-methylenedioxy-9-(2-(adamantan-1-yl) acetoxy)-10-methoxyberberine chloride (Wang et al., 2018). BBR derivatives with a long alkyl chain branched by hydroxyl and methoxycarbonyl groups at position 9 showed 3.6-fold higher intracellular concentrations and 60-fold increased anti-proliferation activity against A549 cells compared with BBR (Liu et al., 2020c). 9-O-gernylberberrubine bromide and 9-O-farnesylberberrubine bromide showed greater growth inhibition, cell cycle regulation, and migration reduction (Chang et al., 2021). Combined with nanotechnology, 9-O-octadecyl substituted BBR derivative induce mitochondrial apoptosis (Song et al., 2019a). 13-dichlorophenylalkyl BBR semisynthetic derivatives, especially 13-[3-(2,4-dichlorophenyl)propyl]-9,10-dimethoxy-5,6-dihy-drobenzo[*g*]-1,3benzodioxolo[5,6-a]quinolizinium chloride induced apoptosis in breast cancer cells, while, 13-[2-(4-chlorophenyl)ethyl]berberine iodide (NAX014) reduced HER2 overexpressing breast cancer cell migration, and intragastric administration of 20 mg/kg NAX014 in HER2/neu transgenic mice delayed the onset of mammary tumors with no negative effects on health and survival (Pierpaoli et al., 2018; Pierpaoli et al., 2021).13-Ethylberberine induces apoptosis through the mitochondria-related apoptotic pathway in radiotherapy-resistant breast cancer cells (Jin et al., 2019). 9-/13-lipophilic substituted BBR derivatives, such as 9-O-dodecyl-BBR, 13-dodecyl-BBR, and 13-O-dodecyl-BBR, show significant photocytotoxic effects on HepG2 cells and induce remarkable cancer cell apoptosis (Lin et al., 2020).

Collectively, although BBR derivatives have higher anticancer efficacy, their specific mechanisms need to be further studied, and preclinical evidence is scarce.

## Strategies to Improve the Bioavailability of BBR

The poor bioavailability of BBR limits its clinical application in cancer treatment. At present, apart from BBR derivatives, several other effective methods have been adopted. Structural modification of BBR not only improves its bioavailability but also enhances its anticancer effects. As mentioned earlier, BBR is sensitive to temperature and is widely distributed in organs after absorption. Evidence indicates that cationic or lipophilic substituted BBR derivatives increase their potential to penetrate the phospholipid bilayer (Popiołek et al., 2019; Lin et al., 2020). Demonstrating good stability at 4°C and 25°C, BBR liposomes showed a sustained-release behavior and displayed significantly increased retention in rat blood circulation, as evidenced by the significantly increased half-life (t_1/2_) and area under the curve (AUC) compared with BBR. In addition, BBR liposomes selectively increased the concentration in the liver, lungs and tumors, while reducing their distribution in the heart and kidney (Wang et al., 2017d). Similarly, the forms of esters, amides, and sulfonates of BBR have also been developed (Wang et al., 2018). In particular, BBR hydrochloride and BBR sulfate have higher solubilities than BBR (Feng et al., 2020). In addition, BBR hydrochloride induced cell proliferation and apoptosis of A549 cells by increasing the activity of the Bcl-2/Bax signaling pathway and inhibiting the Janus kinase 2 (Jak2)/vascular endothelial growth factor (VEGF)/NF-κB/transcription factor AP-1 (AP-1) signaling pathway (Li et al., 2018b). Second, co-administration can significantly improve the bioavailability and anticancer effects of BBR, such as theophylline (Hashemi-Niasari et al., 2018), cinnamaldehyde (Meng et al., 2017), icotinib (Chen et al., 2021), evodiamine (Du et al., 2017), HMQ1611 (Dai et al., 2019), s-allyl-cysteine (Sengupta et al., 2017), resveratrol (D’arcy et al., 2021), and Dox (Zhang et al., 2020b). All these factors may have a synergistic effect with BBR. Furthermore, the advent of nanotechnology has led to considerable improvements in enhancing the targeting and efficacy of BBR. For example, nanofabrication methods generate BBR-loaded liposomes with uniform size, high entrapment efficiency, and extended drug release time (Duong et al., 2021). Collagen gold nanoparticulate nanocarriers conjugated with BBR showed greater cell uptake capacity and pro-apoptotic effect on breast cancer cells (Chiu et al., 2021). Similarly, BBR-loaded Janus gold mesoporous silica nanocarriers were used in chemo/radio/photothermal therapy for the treatment of liver cancer and had a protective effect on normal tissues (Li et al., 2019b). The fluorescence of BBR-loaded polylactic acid nanoparticles (NPs) taken up by HCT116 colon carcinoma cells was approximately 2-fold higher than that of BBR (Ghaffarzadegan et al., 2020). Nano-co-delivery can further improve these effects. Nanoliposomes loaded with P-gp inhibitors and BBR chloride significantly increased BBR chloride content in cells (Vanti et al., 2021). Nano-co-delivery of BBR and Dox using poly lactic-co-glycolic acid NPs resulted in an almost 14-fold increase in half-life and an increased plasma concentration in rats (Khan et al., 2019), while BBR and zinc oxide-based NPs provided safe chemo-photothermal therapy for lung cancers (Kim et al., 2018b). Dual-functionalized spray-dried casein micelles combined with BBR and diosmin showed superior cytotoxicity and higher cellular uptake in HepG2 cells (Abdelmoneem et al., 2018). Targeting the lung delivery of layer-by-layer lipid NPs for the dual delivery of BBR and rapamycin (RAP) enhanced the sensitivity of lung cancer cells to RAP and the toxicity of drugs to cancer cells (Kabary et al., 2018). With BBR mitochondrial targeting, a paclitaxel-ss-BBR conjugate (PTX-ss-BBR NPs) and camptothecin-ss-BBR were prepared, which had stronger anti-A549 cell proliferation and pro-apoptotic effects (Cheng et al., 2020; Cheng and Ji, 2020). In addition, BBR derivatives and Dox nanomedicines target mitochondria-enhanced apoptosis (Lin et al., 2021). Dendrimer-encapsulated BBR improved pharmacokinetic parameters, such as AUC and half-life (t_1/2_) of BBR (Gupta et al., 2017). The gram-scale production of carrier-free fluorescent BBR microrods exhibited good optical properties and, pH-responsive drug-release behavior (Zheng et al., 2018b).

Therefore, the enhancement of BBR bioavailability primarily includes derivatizations, modifications, co-administration, and nanotechnology.

## Conclusion and Future Perspectives

Lung cancer, breast cancer, and liver cancer are common types of cancers globally with a rising incidence rate. Conventional cancer therapies may lead to drug resistance and serious toxicities. Thus, novel therapeutic approaches are urgently needed. Natural products are a valuable source of new drugs, and BBR is a known isoquinoline alkaloid with outstanding activities. It can be extracted from various medicinal plants, such as *C. chinensis*, *P. chinense,* and *B. vulgaris*. In recent decades, accumulated studies have shown that BBR has considerable anticancer effects. As mentioned above, BBR can significantly inhibit the proliferation, metastasis, and invasiveness of cancer cells to induce apoptosis, the related signal pathways are AMPK, MAPK, and AKT pathways. This article reviews the chemical properties, ADMET, therapeutic effects and mechanisms of BBR in treating three types of cancers (breast, lung and liver cancer). Which are expected to serve as a reference for follow-up research.

However, there are some issues require further clarification in future research before clinical usage of this natural compound. Firstly, due to multi-target characteristics, the anticancer mechanisms of BBR have not yet been fully elucidated, and more verification is still needed, for example, study shows BBR regulates immunity during cancer treatment, but its targets are not fully clarified, more research is needed in the future. By means of network pharmacology, research has demonstrated that traditional medicines show a synergistic effect through multiple targets and pathways (Yuan et al., 2017). Therefore, we suggest to search for possible targets of BBR and to find drugs or natural products that have a synergistic effect with BBR through network pharmacology.

Secondly, most of the current existing researches focus on the cellular level, there is still a lack of corresponding clinical data to evaluate anticancer efficacy and corresponding dose in humans, which should be studied further.

Thirdly, the pharmacokinetics of BBR in humans have not been fully elucidated. The metabolites of BBR in the body also need to be further studied. A previous study identified 96 metabolites in rats, with most metabolites excreted in urine (Wang et al., 2017b). However, how metabolites interact with BBR requires further research to better understand their biological activities. Therefore, we recommend more *in vivo* studies to understand the metabolism of BBR.

Fourthly, low bioavailability of BBR seriously limits its clinical application, fortunately, structural modification of BBR has a significant effect on improving efficacy, selectivity, and safety. Some BBR derivatives, such as position 9 or 13 of the alkaloid skeleton, have better bioavailability and anticancer effects, which have been predicted to improve clinical efficacy and safety of BBR (Zhang et al., 2019a). Besides, nanotechnology have significantly enhanced the bioavailability of BBR, also, nanotechnology enables BBR to reach specific sites and exert pharmacological effects. Moreover, nanocarriers can enhance the anticancer effects of BBR through co-delivery. Therefore, a combination of structural modification, BBR derivatives, and nanotechnology is expected to be a comprising way to improve bioavailability of BBR.

Fifthly, regarding safety, as described herein, toxicity of BBR depends on the method of administration and mild gastrointestinal reactions may occur in some patients after oral administration (Imenshahidi and Hosseinzadeh, 2019). Paradoxically, BBR has shown adverse effects and even toxicity under specific circumstances, such as liver toxicity, cardiotoxicity and immunotoxicity. But BBR has been shown to possess cardioprotective, hepatoprotective effects and immunomodulatory capacities in human body. Such contradictory results may be related to mode of administration, dose of BBR and administration time. Hence, we suggest comprehensive strategies to investigate the balance between toxicity and therapeutic efficacy of BBR. Especially, long-term clinical trials are needed to better determine the human safety of BBR.

Sixthly, recently, clinical research on BBR has increased, primarily focusing on metabolic diseases, such as nonalcoholic liver disease, diabetes, and hyperlipidemia (Li et al., 2019a; Zhang et al., 2020c; Di Bisceglie et al., 2020; Zhao et al., 2021b; Harrison et al., 2021), and digestive system diseases, such as ulcerative colitis (Xu et al., 2020) and colorectal adenoma (Chen et al., 2020). BBR ursodeoxycholate and BBR chloride are involved in this process. They provide reliable evidence for the safety and effectiveness of the clinical use of BBR, but a lack of high-quality clinical research on cancers remains. Furthermore, most research is limited to Chinese studies, which may not be appliance for other groups of people. So large sample and multi-center studies are recommended.

Lastly, multidrug resistance has become a challenge in anticancer therapy and BBR has been valued as a chemosensitizer, chemoprotector and radiotherapy protector. Therefore, the sensitization effect of BBR on radiotherapy and chemotherapy warrants further study.

In conclusion, many studies have revealed anticancer effects of BBR through apoptosis, and more *in vivo* and *in vitro* studies are needed to further confirm anticancer effects of BBR. Hopefully, with further research, the therapeutic effect of BBR on cancers will likely be clinically accepted and applied.
